# Supplementary material for: Reported sleep duration reveals segmentation of the adult life-course into three phases
Source: Nat Commun. 2022 Dec 13;13:7697. doi: 10.1038/s41467-022-34624-8 (PMC9744828; doi:10.1038/s41467-022-34624-8)
Supplement: Supplementary file 1 — Supplementary Information [file 41467_2022_34624_MOESM1_ESM.pdf]

# Supplementary Information: Reported sleep duration reveals segmentation of the adult life-course into three phases

A. Coutrot[1*], A. S. Lazar[2], M. Richards[3], E. Manley[4],
J. M. Wiener[5], R. C. Dalton[6], M. Hornberger[2], H. J. Spiers[7*]

[1]LIRIS - CNRS - University of Lyon, France
[2]Norwich Medical School, University of East Anglia, Norwich, United Kingdom
[3]Unit for Lifelong Health and Ageing, University College London, United Kingdom.
[4]School of Geography, University of Leeds, United Kingdom
[5]Department of Psychology, Bournemouth University, United Kingdom
[6]School of Architecture, Lancaster University, Lancaster, United Kingdom
[7]Institute of Behavioural Neuroscience, University College London, United Kingdom.

[*]To whom correspondence should be addressed: antoine.coutrot@cnrs.fr and h.spiers@ucl.fr

## Supplementary Figures

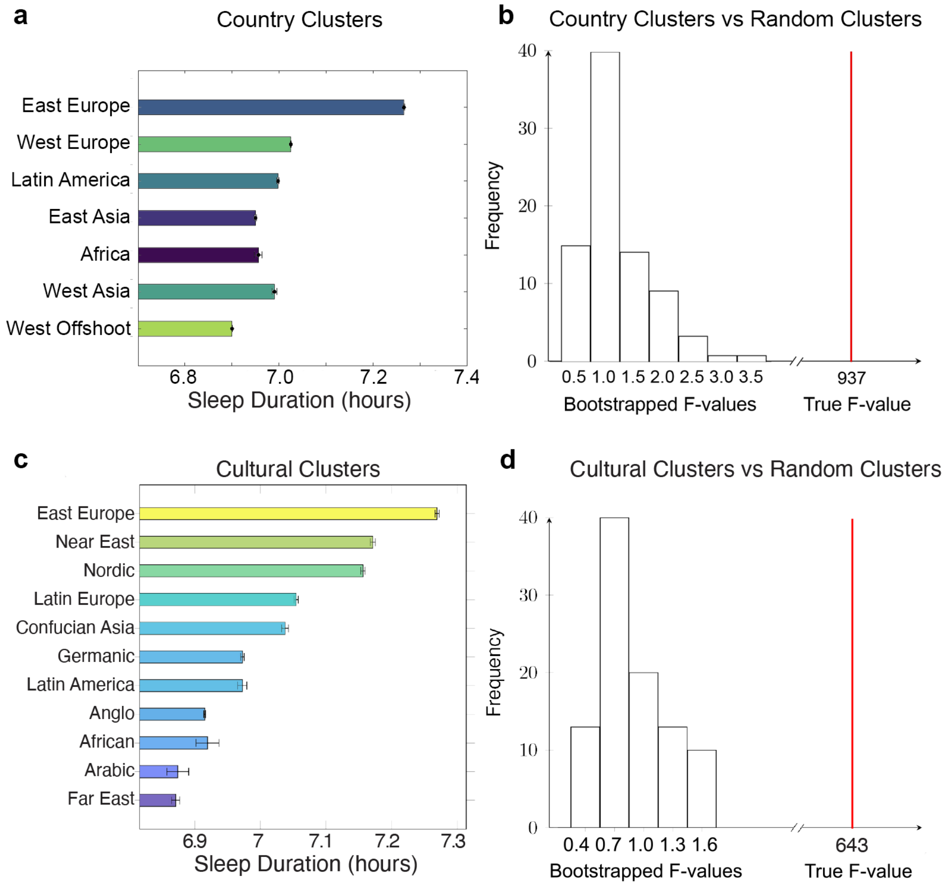


Supplementary Fig. 1: **Between-country sleep duration differences can be clustered into supra-national regions. a -** Average sleep duration in the 7 global clusters of countries defined in (*1*). These clusters are based on historical and economical proximity and constitute one of the most common world map segmentation [25]. The sample size and demographics of these clusters are available Fig. S3. Error bars correspond to 95% confidence intervals. **b -** We randomly shuffled the country labels 100 times. At each iteration, we computed the F-value from an ANOVA predicting the sleep durations from the age, gender, and global clusters based on the random country labels. We show the histogram of the bootstrapped random global clusters F-values (M=0.95, 95%CI=[0.84 1.06]) and the true F-value corresponding to the actual global clusters (vertical red line, F(6, 730181)=936.97), far above the largest random F-value. **c-d -** We repeated this analysis with a slightly different set of clusters, based on cultural similarities between countries (*2*) and obtained similar results.

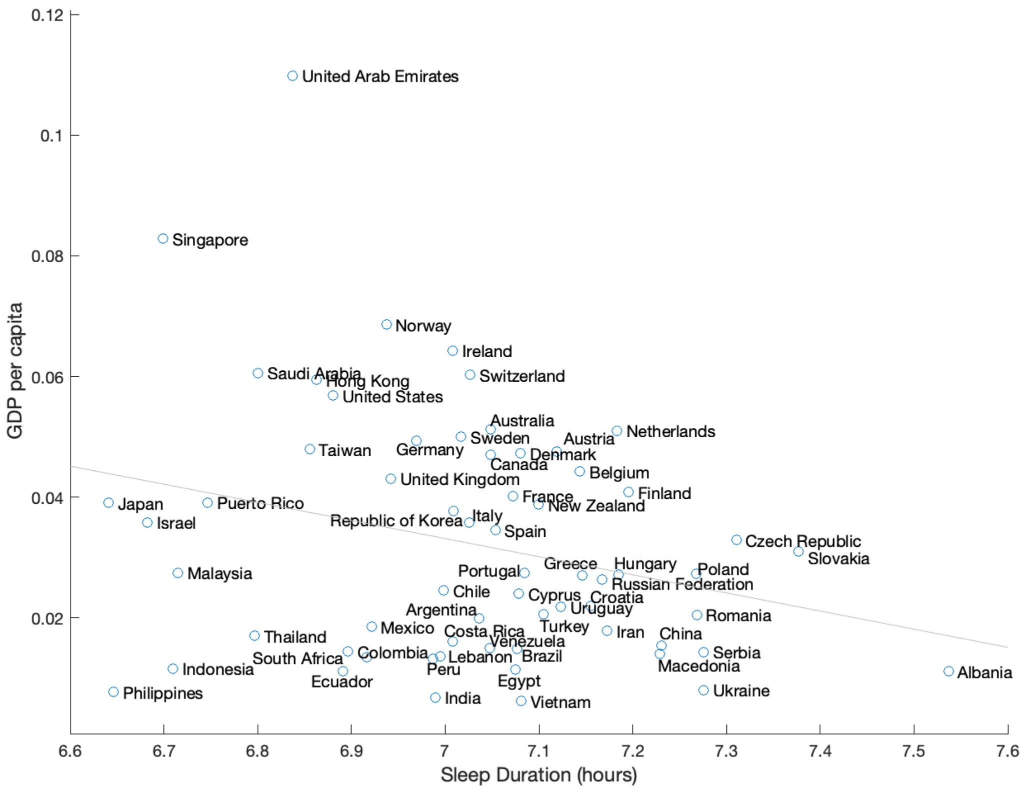


Supplementary Fig. 2: **Association between country-level average sleep duration and Gross Domestic Product per capita.** A linear regression with sleep duration as the response variable gives significant effects for both GDP per capita ($t(60) = -4.57$, $p < 0.001$) and latitude ($t(60) = 6.59$, $p < 0.001$). We computed the same regression with the countries' conditional modes (country-level deviation from the population-level average predicted sleep duration corrected for the other demographics) as predictors, and also found a significant effect of both GDP per capita ($t(60) = -2.37$, $p = 0.02$) and latitude ($t(60) = 3.38$, $p = 0.001$)).

**a** - Country clusters defined in Maddison (2010), based on historical and economic proximity

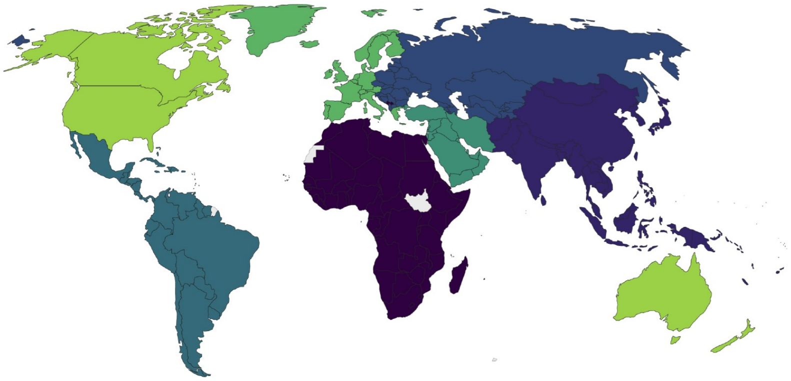

| Country Cluster | N | Males | Age ($M \pm SD$) | Sleep Duration ($M \pm SD$) |
|---|---|---|---|---|
| East Europe | 77296 | 43312 | 30.96 (±11.04) | 7.26 (±1.16) |
| West Europe | 342692 | 185087 | 40.39 (±14.25) | 7.02 (±1.01) |
| Latin America | 36219 | 20909 | 33.65 (±13.29) | 6.99 (±1.13) |
| East Asia | 38005 | 22879 | 29.60 (±10.20) | 6.95 (±1.08) |
| Africa | 2763 | 1675 | 37.12 (±14.38) | 6.95 (±1.12) |
| West Asia | 8771 | 5987 | 33.16 (±11.13) | 6.99 (±1.15) |
| West Offshoot | 224441 | 101304 | 41.39 (±15.15) | 6.90 (±1.05) |

**b** - Country clusters defined in Ronen & Shenkar (2013), based on cultural proximity

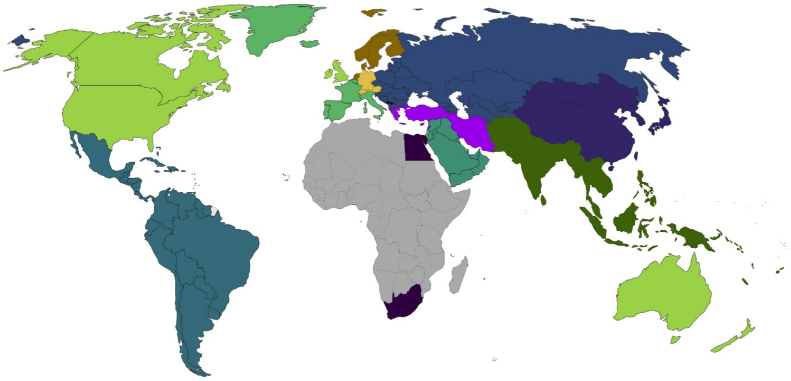

| Cultural Cluster | N | Males | Age ($M \pm SD$) | Sleep Duration ($M \pm SD$) |
|---|---|---|---|---|
| East Europe | 77296 | 43312 | 30.96 (±11.04) | 7.26 (±1.16) |
| Near East | 46584 | 27888 | 34.05 (±11.19) | 7.14 (±1.16) |
| Nordic | 51742 | 24310 | 38.00 (±14.04) | 7.15 (±0.99) |
| Latin Europe | 59642 | 34851 | 37.28 (±13.93) | 7.04 (±1.00) |
| Confucian Asia | 21205 | 11165 | 30.57 (±10.20) | 7.03 (±1.01) |
| Latin America | 36219 | 20909 | 33.65 (±13.29) | 6.99 (±1.13) |
| Germanic | 71762 | 38587 | 42.39 (±13.90) | 6.97 (±0.98) |
| Anglo | 343869 | 165154 | 42.24(±14.95) | 6.91 (±1.03) |
| African | 2763 | 1675 | 37.12 (±14.38) | 6.95 (±1.12) |
| Arabic | 2305 | 1588 | 34.01 (±11.35) | 6.81 (±1.19) |
| Far East | 16800 | 11714 | 28.37 (±10.07) | 6.83 (±1.15) |

Supplementary Fig. 3: **a -** Demographics of the supra-national clusters defined in (*1*). **b -** Demographics of the supra-national clusters defined in (*2*).

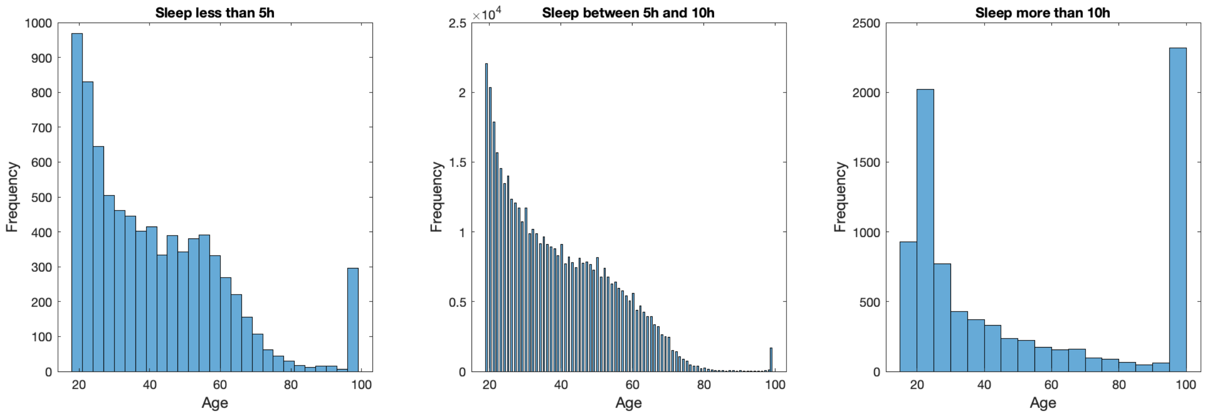


Supplementary Fig. 4: Age distribution of participants who reported sleeping less than 5h, between 5h and 10h, more than 10h.

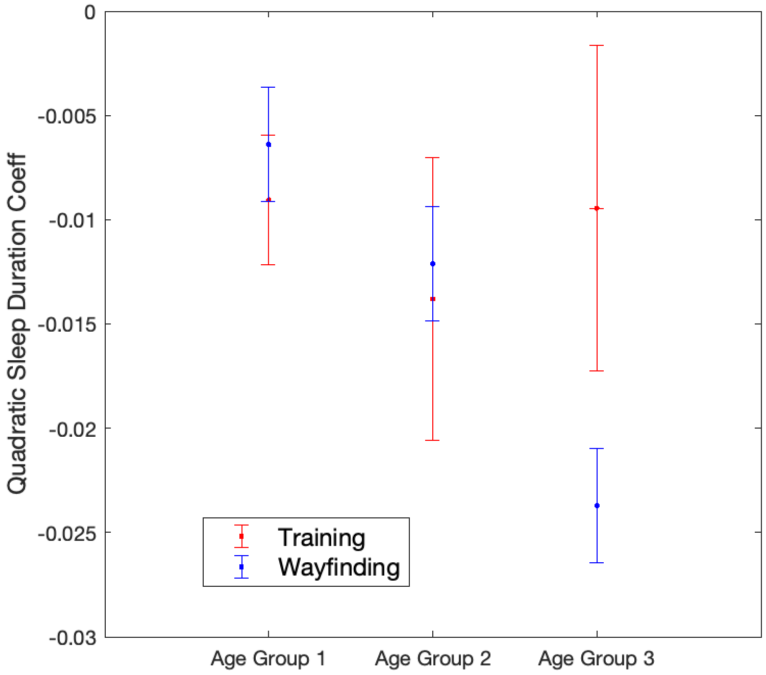


Supplementary Fig. 5: Quadratic sleep duration coefficients from 3 ANOVAs independently computed for each age group identified in Fig. 1, with Wayfinding Performance and Training Performance as the response variable. The structure of the tested models is ‘performance $\sim$ sleepduration + sleepduration$^2$ + education + home environment + commute duration’. Error bars correspond to 95% confidence intervals.

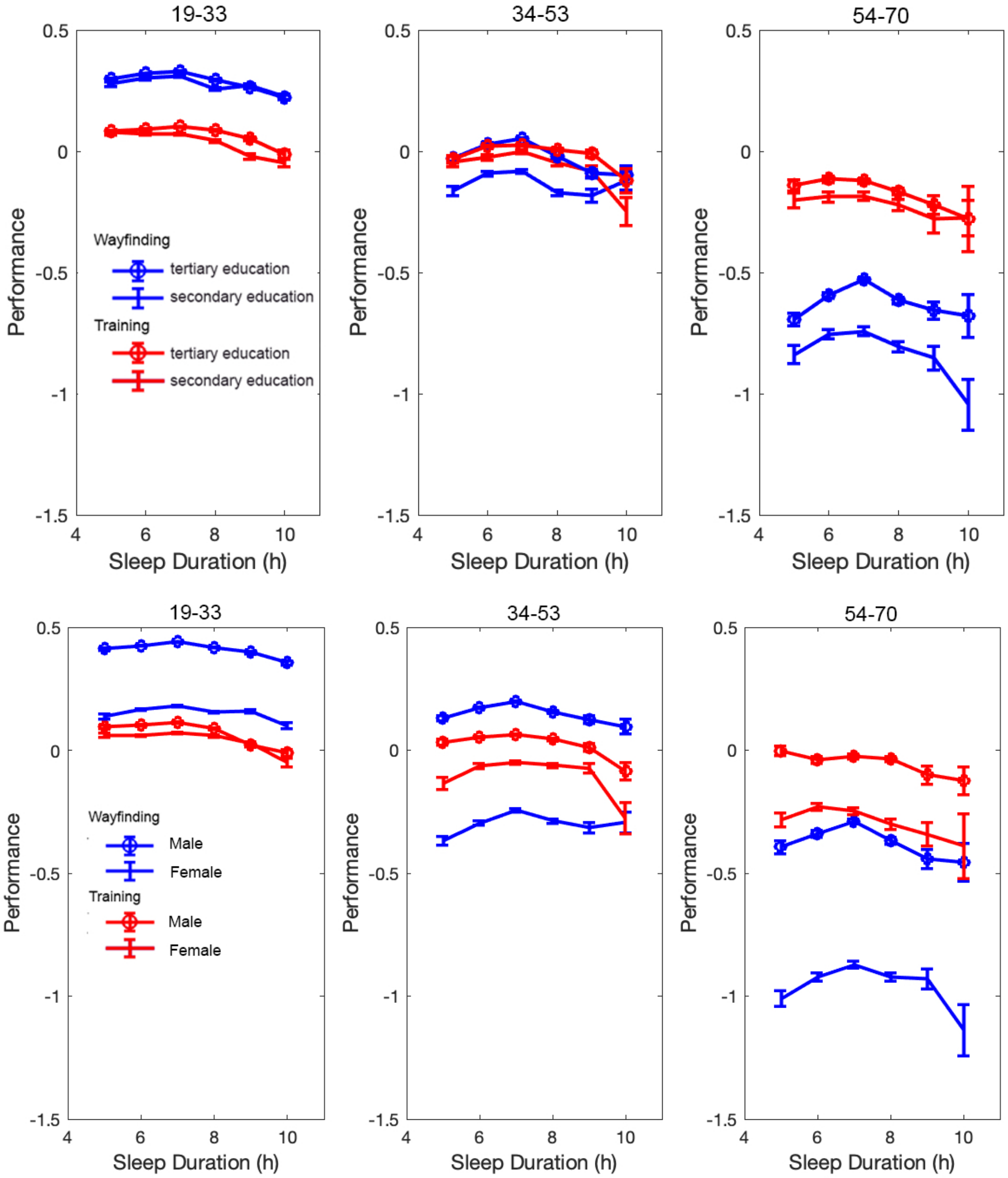


Supplementary Fig. 6: **Association between reported sleep duration and spatial ability in the 3 age groups identified in Fig. 1, stratified by level of education (top) and gender (bottom).** Spatial ability was quantified with the Sea Hero Quest (SHQ) wayfinding task. Training levels did not require spatial ability as the target was visible from the starting point. In the first, second and third age groups, the sample sizes are respectively n = 321,406, n = 263,932 and n = 144,849. Error bars correspond to standard errors, their centers correspond to the mean performance for a given sleep duration.

# Supplementary Tables

**Table S1 -** Country-level Characteristics.

| Country | 2L | isWEIRD | Mean Sleep (h) | Means Sleep Under 40 (h) | GDP per Capita | Mean Latitude | Sample Size |
|---|---|---|---|---|---|---|---|
| United Arab Emirates | AE | 0 | 6.84 | 6.8769 | 0.10987 | 24.2578 | 1376 |
| Albania | AL | 0 | 7.5367 | 7.5484 | 0.011122 | 41.0911 | 941 |
| Argentina | AR | 0 | 7.0364 | 7.0955 | 0.019854 | -37.667 | 3684 |
| Austria | AT | 1 | 7.1185 | 7.2132 | 0.047587 | 47.5748 | 3477 |
| Australia | AU | 1 | 7.0488 | 7.1427 | 0.051178 | -25.2184 | 16134 |
| Belgium | BE | 1 | 7.1437 | 7.197 | 0.044259 | 50.5956 | 3869 |
| Brazil | BR | 0 | 7.0766 | 7.0914 | 0.014859 | -9.8028 | 14089 |
| Canada | CA | 1 | 7.0485 | 7.0667 | 0.046991 | 65.2941 | 21732 |
| Switzerland | CH | 1 | 7.0269 | 7.0721 | 0.060258 | 46.7416 | 5880 |
| Chile | CL | 0 | 6.9982 | 7.0266 | 0.024515 | -44.8384 | 3269 |
| China | CN | 0 | 7.2304 | 7.2549 | 0.015327 | 35.3034 | 11482 |
| Colombia | CO | 0 | 6.8962 | 6.9316 | 0.014424 | 3.4711 | 2735 |
| Costa Rica | CR | 0 | 7.0079 | 7.0013 | 0.016076 | 9.7459 | 1133 |
| Cyprus | CY | 0 | 7.0784 | 7.2241 | 0.023953 | 34.9549 | 523 |
| Czech Republic | CZ | 0 | 7.3105 | 7.3726 | 0.032872 | 49.8784 | 24423 |
| Germany | DE | 1 | 6.9695 | 7.0495 | 0.049371 | 51.1662 | 64569 |
| Denmark | DK | 1 | 7.0804 | 7.131 | 0.047236 | 55.7047 | 3046 |
| Ecuador | EC | 0 | 6.8912 | 6.919 | 0.011196 | -1.6431 | 726 |
| Egypt | EG | 0 | 7.0745 | 7.1297 | 0.011387 | 27.6394 | 738 |
| Spain | ES | 1 | 7.0534 | 7.1738 | 0.034519 | 38.5314 | 14542 |
| Finland | FI | 1 | 7.1953 | 7.191 | 0.040817 | 64.3201 | 1157 |
| France | FR | 1 | 7.0726 | 7.1506 | 0.04022 | 35.085 | 15257 |
| United Kingdom | GB | 1 | 6.9418 | 7.0581 | 0.043045 | 55.5618 | 121983 |
| Greece | GR | 0 | 7.1465 | 7.2571 | 0.026977 | 38.3598 | 41247 |
| Hong Kong | HK | 0 | 6.8631 | 6.8773 | 0.059431 | 22.3726 | 2031 |
| Croatia | HR | 0 | 7.1551 | 7.2893 | 0.021957 | 44.64 | 1283 |
| Hungary | HU | 0 | 7.1846 | 7.2519 | 0.027165 | 47.2323 | 16504 |
| Indonesia | ID | 0 | 6.7095 | 6.7173 | 0.01162 | -2.9963 | 2200 |
| Ireland | IE | 1 | 7.0078 | 7.098 | 0.064257 | 53.4979 | 2708 |
| Israel | IL | 0 | 6.6822 | 6.7487 | 0.035784 | 31.8831 | 925 |
| India | IN | 0 | 6.9893 | 7.014 | 0.006803 | 23.3842 | 6551 |
| Iran | IR | 0 | 7.1723 | 7.2302 | 0.017788 | 33.2924 | 1764 |
| Italy | IT | 1 | 7.0259 | 7.1645 | 0.035743 | 42.1826 | 21976 |
| Japan | JP | 0 | 6.6409 | 6.7361 | 0.039003 | 35.4739 | 621 |
| Republic of Korea | KR | 0 | 7.0085 | 6.9966 | 0.03769 | 35.7337 | 1059 |
| Lebanon | LB | 0 | 6.9947 | 7.1158 | 0.01367 | 33.8606 | 566 |
| Macedonia | MK | 0 | 7.2292 | 7.3264 | 0.014032 | 41.6354 | 637 |
| Mexico | MX | 0 | 6.9214 | 6.9523 | 0.018519 | 24.2179 | 7954 |
| Malaysia | MY | 0 | 6.7151 | 6.7366 | 0.0275 | 3.9696 | 2355 |
| Netherlands | NL | 1 | 7.1834 | 7.2956 | 0.05097 | 48.2584 | 43215 |
| Norway | NO | 1 | 6.9372 | 7.0079 | 0.068552 | 69.8766 | 1989 |
| New Zealand | NZ | 1 | 7.0998 | 7.2005 | 0.038756 | -40.8627 | 3305 |
| Peru | PE | 0 | 6.9864 | 7.0141 | 0.013223 | -7.4457 | 959 |
| Philippines | PH | 0 | 6.6462 | 6.6644 | 0.0076916 | 11.4573 | 3050 |
| Poland | PL | 0 | 7.268 | 7.308 | 0.027342 | 51.4007 | 14602 |
| Puerto Rico | PR | 0 | 6.7466 | 6.7561 | 0.039083 | 18.1731 | 959 |
| Portugal | PT | 1 | 7.0848 | 7.1409 | 0.027409 | 39.2181 | 4349 |
| Romania | RO | 0 | 7.2682 | 7.3014 | 0.020483 | 45.8446 | 5247 |
| Serbia | RS | 0 | 7.2757 | 7.3569 | 0.014316 | 44.2476 | 602 |
| Russian Federation | RU | 0 | 7.1676 | 7.1705 | 0.026325 | 63.3803 | 5067 |
| Saudi Arabia | SA | 0 | 6.8002 | 6.8428 | 0.060584 | 23.1493 | 961 |
| Sweden | SE | 1 | 7.017 | 7.1119 | 0.050008 | 61.8845 | 3241 |
| Singapore | SG | 0 | 6.6994 | 6.718 | 0.08285 | 1.3664 | 2828 |
| Slovakia | SK | 0 | 7.3768 | 7.4392 | 0.030996 | 48.8382 | 7012 |
| Thailand | TH | 0 | 6.7971 | 6.7627 | 0.01697 | 13.2487 | 1700 |
| Turkey | TR | 0 | 7.1049 | 7.1264 | 0.020657 | 39.0661 | 3288 |
| Taiwan | TW | 0 | 6.8553 | 6.88 | 0.04794 | 23.7266 | 3310 |
| Ukraine | UA | 0 | 7.2754 | 7.2852 | 0.0080075 | 48.7207 | 1180 |
| United States | US | 1 | 6.8808 | 6.8887 | 0.056823 | 48.7409 | 193210 |
| Uruguay | UY | 0 | 7.1237 | 7.1567 | 0.0218 | -32.2973 | 582 |
| Venezuela | VE | 0 | 7.0478 | 7.1511 | 0.014969 | 7.3982 | 753 |
| Vietnam | VN | 0 | 7.0814 | 7.0876 | 0.0061866 | 16.5251 | 1081 |
| South Africa | ZA | 0 | 6.9165 | 6.97 | 0.013477 | -28.8461 | 2133 |

Table. S 1: Demographics of the 63 countries included in the analysis.

| Fixed Effects | Estimate (95% CI) | Standard Error | t-value | p-value |
|---|---|---|---|---|
| Intercept | 8.44 (8.39 8.49) | 0.026 | 323.88 | $< 0.001$ |
| Age | -0.060 (-0.061 -0.059) | $6.0 \times 10^{-4}$ | -98.95 | $< 0.001$ |
| Age$^2$ | $6.1 \times 10^{-4}$ (6.0 6.2)$10^{-4}$ | $6.6 \times 10^{-6}$ | 92.80 | $< 0.001$ |
| Gender | -0.18 (-0.19 -0.17) | $6.9 \times 10^{-3}$ | -26.00 | $< 0.001$ |
| Commute | -0.29 (-0.31 -0.27) | $9.2 \times 10^{-3}$ | -31.32 | $< 0.001$ |
| Education | 0.19 (0.18 0.21) | $7.9 \times 10^{-3}$ | 25.00 | $< 0.001$ |
| Home Environment | -0.063 (-0.078 -0.049) | $7.7 \times 10^{-3}$ | -8.15 | $< 0.001$ |
| Age*Gender | $1.6 \times 10^{-3}$ (1.3 1.9)$10^{-3}$ | $1.7 \times 10^{-4}$ | 9.55 | $< 0.001$ |
| Age*Commute | $1.5 \times 10^{-3}$ (1.1 2.0)$10^{-3}$ | $2.3 \times 10^{-4}$ | 6.72 | $< 0.001$ |
| Age*Education | $-4.9 \times 10^{-3}$ (-5.2 -4.5)$10^{-3}$ | $1.8 \times 10^{-4}$ | -26.55 | $< 0.001$ |
| Age*Home Environment | $2.2 \times 10^{-3}$ (1.8 2.6)$10^{-3}$ | $1.9 \times 10^{-4}$ | 11.84 | $< 0.001$ |
| Random Effect | Std (95% CI) | VPC (95% CI) | | |
| Country (intercept) | 0.17 (0.16 0.21) | 2.75% (2.02 3.81) | | |
| Error (intercept) | 1.04 (1.03 1.04) | | | |
| sample size = 729,948, 63 countries | | | | |

Table. S 2: Output of the linear mixed model calculated to predict sleep duration with age, age$^2$, gender, education, home environment, commute duration and their interaction with age as fixed effects, and random intercepts clustered by countries: sleep duration $\sim$ age*(gender + education + home environment + commute duration) + age$^2$ + (1 $|$ country).

| | Wayfinding Performance | | Training Performance | |
|---|---|---|---|---|
| Variable | F-value | p-value | F-value | p-value |
| Intercept | 451.86 | $< 0.001$ | 58.08 | $< 0.001$ |
| Gender | 19918 | $< 0.001$ | 1161.90 | $< 0.001$ |
| SleepDuration | 186.16 | $< 0.001$ | 41.45 | $< 0.001$ |
| SleepDuration$^2$ | 193.97 | $< 0.001$ | 49.58 | $< 0.001$ |
| AgeGroups | 136.32 | $< 0.001$ | 6.03 | 0.002 |
| Commute | 11.73 | $< 0.001$ | 3.97 | 0.02 |
| Education | 961.01 | $< 0.001$ | 166.26 | $< 0.001$ |
| Home Environment | 1452.5 | $< 0.001$ | 178.88 | $< 0.001$ |
| AgeGroups*SleepDuration | 49.56 | $< 0.001$ | 2.40 | 0.09 |
| AgeGroups*SleepDuration$^2$ | 55.05 | $< 0.001$ | 3.19 | 0.04 |
| sample size = 418,152 | | | | |

Table. S 3: Output of two ANOVAs calculated to predict wayfinding and training performance with sleepduration, sleepduration$^2$ and their interaction with age groups, gender, education, home environment, commute duration as independent variables: performance $\sim$ agegroups*(sleepduration +sleepduration$^2$) + education + home environment + commute duration. The 3 age groups are the ones identified in Fig 1C: younger (19-33 y.o., N=191,791), middle (34-53 y.o., N=153,884), and older (54-70 y.o., N=72,477) participants.

## Supplementary References


1. A. Maddison, *The world economy*. OECD publishing, 2006.

2. S. Ronen and O. Shenkar, "Mapping world cultures: Cluster formation, sources and implications," *Journal of International Business Studies*, vol. 44, no. 9, pp. 867–897, 2013.
