# Supplementary material for: Reported sleep duration reveals segmentation of the adult life-course into three phases
Source: Nat Commun. 2022 Dec 13;13:7697. doi: 10.1038/s41467-022-34624-8 (PMC9744828; doi:10.1038/s41467-022-34624-8)
Supplement: Supplementary file 3 — Reporting Summary [file 41467_2022_34624_MOESM3_ESM.pdf]

nature portfolio

Corresponding author(s): Antoine Coutrot
Hugo Spiers

Last updated by author(s): 03/06/2022

Reporting Summary

Nature Portfolio wishes to improve the reproducibility of the work that we publish. This form provides structure for consistency and transparency in reporting. For further information on Nature Portfolio policies, see our Editorial Policies and the Editorial Policy Checklist.

Statistics

For all statistical analyses, confirm that the following items are present in the figure legend, table legend, main text, or Methods section.

n/a	Confirmed	
☐	☒	The exact sample size (*n*) for each experimental group/condition, given as a discrete number and unit of measurement
☐	☒	A statement on whether measurements were taken from distinct samples or whether the same sample was measured repeatedly
☐	☒	The statistical test(s) used AND whether they are one- or two-sided<br>*Only common tests should be described solely by name; describe more complex techniques in the Methods section.*
☐	☒	A description of all covariates tested
☐	☒	A description of any assumptions or corrections, such as tests of normality and adjustment for multiple comparisons
☐	☒	A full description of the statistical parameters including central tendency (e.g. means) or other basic estimates (e.g. regression coefficient) AND variation (e.g. standard deviation) or associated estimates of uncertainty (e.g. confidence intervals)
☐	☒	For null hypothesis testing, the test statistic (e.g. *F*, *t*, *r*) with confidence intervals, effect sizes, degrees of freedom and *P* value noted<br>*Give P values as exact values whenever suitable.*
☒	☐	For Bayesian analysis, information on the choice of priors and Markov chain Monte Carlo settings
☐	☒	For hierarchical and complex designs, identification of the appropriate level for tests and full reporting of outcomes
☐	☒	Estimates of effect sizes (e.g. Cohen's *d*, Pearson's *r*), indicating how they were calculated

Our web collection on statistics for biologists contains articles on many of the points above.

Software and code

Policy information about availability of computer code

Data collection	The data was collected via Sea Hero Quest, a video game using Unity 3D (Unity Technologies, Copenhagen Denmark) for smart phones and tablets (apple and android devices). 'Sea Hero Quest' (SHQ) was released on 4 May 2016 on the App Store for iOS and on Google Play for Androids. If the participant agreed, their data were anonymously stored in a secure T-Systems server in Germany. The application is managed by T-Systems' scalable Docker offering called 'AppAgile' which is operated out of T-Systems' datacenter to ensure data integrity and data privacy according to German data security law.
Data analysis	Matlab 2018a

For manuscripts utilizing custom algorithms or software that are central to the research but not yet described in published literature, software must be made available to editors and reviewers. We strongly encourage code deposition in a community repository (e.g. GitHub). See the Nature Portfolio guidelines for submitting code & software for further information.

Data

Policy information about availability of data

All manuscripts must include a data availability statement. This statement should provide the following information, where applicable:
- Accession codes, unique identifiers, or web links for publicly available datasets
- A description of any restrictions on data availability
- For clinical datasets or third party data, please ensure that the statement adheres to our policy

The data and Matlab code (R2018a) necessary to reproduce the results presented in this manuscript are available at https://osf.io/j7sd4/?view_only=92c1aa56abfc4d0f99501c9f260fa324
We also set up a portal where researchers can invite a targeted group of participants to play Sea Hero Quest and generate data about their spatial navigation

capabilities. Those invited to play the game will be sent a unique participant key, generated by the Sea Hero Quest system according to the criteria and requirements of a specific project. https://seaheroquest.alzheimersresearchuk.org/ Access to the portal will be granted for non commercial purposes.

Field-specific reporting

Please select the one below that is the best fit for your research. If you are not sure, read the appropriate sections before making your selection.

☒ Life sciences ☐ Behavioural & social sciences ☐ Ecological, evolutionary & environmental sciences

For a reference copy of the document with all sections, see nature.com/documents/nr-reporting-summary-flat.pdf

Life sciences study design

All studies must disclose on these points even when the disclosure is negative.

Sample size	One of the goal of the Sea Hero Quest project was to constitute a worldwide database of how people navigate. We aimed to collect as many participants from as many country as possible. We collected 3.9 million participants from every country in the world.
Data exclusions	A total of 3,881,449 participants played at least one level of the game. 60.8% of the participants provided basic demographics (age, gender, home country) and 27.6% provided more detailed demographics (home environment, level of education). We removed participants above 70 years old because we have previously shown that they suffer from a strong selection bias, causing their performance to be substantially higher than would be expected in unselected participants of the same age \cite{Coutrot2018dt}. We removed participants with an average sleep duration above 10h (0.6%) or below 5h (0.9%). To ensure robust results within countries, we removed participants from countries with fewer than 500 players. This resulted in 730,187 participants from 63 countries included in our analysis, (see Table S1). To provide a reliable estimate of spatial navigation ability, we examined the data from a subset of participants who completed at least the first four wayfinding levels (N = 418,152).
Replication	N/A
Randomization	Participants were not assigned to different groups, they all completed the same task.
Blinding	See above: no group allocation was required in this study.

Reporting for specific materials, systems and methods

We require information from authors about some types of materials, experimental systems and methods used in many studies. Here, indicate whether each material, system or method listed is relevant to your study. If you are not sure if a list item applies to your research, read the appropriate section before selecting a response.

Materials & experimental systems

n/a	Involved in the study	
☒	☐	Antibodies
☒	☐	Eukaryotic cell lines
☒	☐	Palaeontology and archaeology
☒	☐	Animals and other organisms
☐	☒	Human research participants
☒	☐	Clinical data
☒	☐	Dual use research of concern

Methods

n/a	Involved in the study	
☒	☐	ChIP-seq
☒	☐	Flow cytometry
☒	☐	MRI-based neuroimaging

Human research participants

Policy information about studies involving human research participants

Population characteristics	Among the 730,187 included participants, 381,153 identified as males and 349,034 as females. The mean age was 38.71 years (SD=14.53 years). 526,170 reported having received a tertiary education and 204,017 a secondary education or less. 222,097 reported having grown up in a city environment and 508,090 outside cities. 291,822 reported commuting on average less than 30 minutes per day, 254,362 between 30 minutes and 1 hour, and 183,764 more than 1 hour.<br>Among the 418,152 participants included for the spatial ability analysis, 224,159 identified as males and 193,993 as females. The mean age was 37.80 years (SD=14.13 years). 301,515 reported having received a tertiary education and 116,637 a secondary education or less. 123,733 reported having grown up in a city environment and 294,419 outside cities. 162,680 reported commuting on average less than 30 minutes per day, 148,167 between 30 minutes and 1 hour, and 107,305 more than 1 hour.
Recruitment	Sea Hero Quest was released on 4 May 2016 on the App Store for iOS and on Google Play for Androids. It was available in 17 languages: English, French, German, Spanish, Macedonian, Greek, Croatian, Dutch, Albanian, Hungarian, Romanian, Slovak, Czech, Polish, Portuguese, Italian, and Serbian. We removed participants above 70 years old because we previously showed a

strong selection bias in this group causing their performance to be substantially higher than would be expected in unselected participants of the same age [10]. This is likely due to the fact that older people using their smarthphone to play videogames are not representative of their age group. However, this bias did not prevent our task to capture spatial ability, as we demonstrated in Coutrot et al., PLoS ONE 2019, by comparing participants' performance at the game and at a similar task in the real world.

Ethics oversight	This study has been approved by UCL Ethics Research Committee. The ethics project ID number is CPB/2013/015

Note that full information on the approval of the study protocol must also be provided in the manuscript.
